# False positive circumsporozoite protein ELISA: a challenge for the estimation of the entomological inoculation rate of malaria and for vector incrimination

**DOI:** 10.1186/1475-2875-10-195

**Published:** 2011-07-18

**Authors:** Lies Durnez, Wim Van Bortel, Leen Denis, Patricia Roelants, Aurélie Veracx, Ho Dinh Trung, Tho Sochantha, Marc Coosemans

**Affiliations:** 1 Insitute of Tropical Medicine, Department of Parasitology, Nationalestraat 155, B-2000 Antwerpen, Belgium; 2 Université de la Méditerranée, Faculté de Médecine, Unité de Recherche en Maladies Infectieuses et Tropicales Emergentes, CNRS-IRD, UMR 6236, boulevard Jean Moulin 27, 13385 Marseille Cedex 5, France; 3 National Institute of Malariology, Parasitology and Entomology, Departement of Entomology, Luong The Vinh Street 245, B.C. 10200 Tu Liem, Hanoi, Vietnam; 4 National Center for Malaria Control, Parasitology and Entomology, Phnom Penh, Cambodia

## Abstract

**Background:**

The entomological inoculation rate (EIR) is an important indicator in estimating malaria transmission and the impact of vector control. To assess the EIR, the enzyme-linked immunosorbent assay (ELISA) to detect the circumsporozoite protein (CSP) is increasingly used. However, several studies have reported false positive results in this ELISA. The false positive results could lead to an overestimation of the EIR. The aim of present study was to estimate the level of false positivity among different anopheline species in Cambodia and Vietnam and to check for the presence of other parasites that might interact with the anti-CSP monoclonal antibodies.

**Methods:**

Mosquitoes collected in Cambodia and Vietnam were identified and tested for the presence of sporozoites in head and thorax by using CSP-ELISA. ELISA positive samples were confirmed by a *Plasmodium *specific PCR. False positive mosquitoes were checked by PCR for the presence of parasites belonging to the Haemosporidia, Trypanosomatidae, Piroplasmida, and Haemogregarines. The heat-stability and the presence of the cross-reacting antigen in the abdomen of the mosquitoes were also checked.

**Results:**

Specimens (N = 16,160) of seven anopheline species were tested by CSP-ELISA for *Plasmodium falciparum *and *Plasmodium vivax *(Pv210 and Pv247). Two new vector species were identified for the region: *Anopheles pampanai *(*P. vivax*) and *Anopheles barbirostris *(*Plasmodium malariae*). In 88% (155/176) of the mosquitoes found positive with the *P. falciparum *CSP-ELISA, the presence of *Plasmodium *sporozoites could not be confirmed by PCR. This percentage was much lower (28% or 5/18) for *P. vivax *CSP-ELISAs. False positive CSP-ELISA results were associated with zoophilic mosquito species. None of the targeted parasites could be detected in these CSP-ELISA false positive mosquitoes. The ELISA reacting antigen of *P. falciparum *was heat-stable in CSP-ELISA true positive specimens, but not in the false positives. The heat-unstable cross-reacting antigen is mainly present in head and thorax and almost absent in the abdomens (4 out of 147) of the false positive specimens.

**Conclusion:**

The CSP-ELISA can considerably overestimate the EIR, particularly for *P. falciparum *and for zoophilic species. The heat-unstable cross-reacting antigen in false positives remains unknown. Therefore it is highly recommended to confirm all positive CSP-ELISA results, either by re-analysing the heated ELISA lysate (100°C, 10 min), or by performing *Plasmodium *specific PCR followed if possible by sequencing of the amplicons for *Plasmodium *species determination.

## Background

The entomological inoculation rate (EIR) is an important indicator in estimating malaria transmission and the impact of vector control. It is defined as the number of infective bites per person per unit of time. In practice, it is calculated by multiplying the average number of bites per person per night with the proportion of infected anophelines (i.e. the sporozoite rate) [1]. This sporozoite rate can be obtained by using different methods. Traditionally, the dissection and microscopic examination of the salivary glands of individual mosquitoes has been used to observe the presence of sporozoites. Although this method is considered as the 'gold standard', it is not practical for assaying a high number of mosquitoes because it is very labour intensive and the samples should be processed freshly. Therefore, other methods have been developed to assess the sporozoite rate. As from the mid-1980s, the enzyme-linked immunosorbent assay (ELISA) using monoclonal antibodies targeting the circumsporozoite protein (CSP) has been increasingly used for estimating the sporozoite rate [2-4]. The antibodies used in the ELISA for detection of *Plasmodium falciparum *and *Plasmodium vivax*, bind to the respective repeat regions [5-7].

The main advantage of ELISA as compared to dissection is the fact that the collected mosquitoes can be stored until processed and the possibility of distinguishing the different human *Plasmodium *species by species-specific monoclonal antibodies. In general, the ELISA technique is less sensitive than dissection, especially when low numbers of sporozoites are present in the salivary glands [8]. Yet, ELISA does not only detect the sporozoites in the salivary glands, but also detects CSP in other mosquito tissues. This results finally in an overestimation of the sporozoite rate, even if only the head-thorax part of the mosquito is used for the ELISA [8,9]. A third technique to detect sporozoites in mosquitoes is *Plasmodium *specific polymerase chain reaction (PCR). Theoretically, PCR should be able to detect 1 sporozoite; in practice *Plasmodium *specific PCR assays can detect as few as 10 sporozoites [10], while ELISA requires at least 100 sporozoites [11]. A disadvantage of PCR is that it will detect the presence of all *Plasmodium *DNA and not only the sporozoites. The ELISA method is stage specific and would be preferred to PCR.

ELISA is nowadays widely used to estimate the sporozoite index. However, Table 1 shows that several studies have reported false ELISA positive results to detect sporozoites in mosquitoes as compared to microscopy or PCR methods [12-15]. The false positive results could lead to an overestimation of the EIR, especially in zoophilic mosquitoes, which can have important implications for estimating malaria transmission, vector incrimination, and the evaluation of vector control strategies. In some studies, this false positivity is being attributed to unidentified factors present in the bovine blood or pig blood, but not in all animals tested [13,15]. These transitory antigens responsible for false positive reactions may than have another origin such as pathogens present in the blood.

**Table 1:** Reported false positivity in CSP-ELISA assays

Country (Reference)	Specimen origin	Number of ELISA positive specimens/Number of specimens tested	Number of specimens confirmed by PCR
Blood specimens tested*			

Thailand [15]	Cow blood	12/16	0
	Pig blood	3/12	0

Senegal [13]	Cow blood	21/56	0

**Mosquito head-thorax specimens tested**			

Gabon [37]	*An. gambiae *s.s.		
	*An. moucheti*	28/1535	18
	*An. funestus*		

South-Africa [12]	*An. demeilloni*	44/unkown	3
	*An. marshalli*	6/unknown	1
	*An. rivulorum*	4/unknown	2

South-Africa [14]	*An. parensis*	20/149	0

Cameroon [36]	*An. gambiae *s.s.		
	*An. funestus*	298/2773	263
	*An. nili*		

* ELISA was performed on cow and/or pig blood because false positivity was suspected in *Anopheles *species that were suspected to have fed on cows or pigs.

In this study, a large dataset of mosquitoes collected in Southeast Asia is presented and tested for the presence of sporozoites by using CSP-ELISA and subsequent confirmation by PCR. The aim of the study was to estimate the level of false positivity among different anopheline species and to check for the presence of other parasites that might interact with the anti-CSP monoclonal antibodies.

## Methods

### Mosquito collection in Cambodia

Two entomological surveys (August-September and November-December 2005) were conducted in twelve forest villages. Six villages were located in the western part and six villages in the eastern part of the country. A more detailed description of the study sites is given in Additional file 1. For each entomological survey, outdoor human landing collections were carried out during six successive nights inside the village and at the forest plot. Human landing collections from 18H till 6H were done by two collectors in each site. The entomological surveys were approved by the ethical committees of the National Centre of Malariology CNM in Phnom Penh (Cambodia) and of the Institute of Tropical Medicine of Antwerp (Belgium).

### Mosquito collection in Vietnam

Mosquito collections in Vietnam were described by Van Bortel *et al *[16]. Briefly, five entomological surveys (November 2004, October and November 2005, October and November 2006) were conducted in three villages in Ma Noi commune and in five villages in Phuoc Binh commune located in the hilly and forested part of Ninh Thuan province. A more detailed description of the study sites is given in Additional file 2. Per study village collections were made inside the village, in the forest and on the way from the village to the forest. Outdoor and indoor human landing collections were made during eight nights per survey.

### Mosquito processing

Adult mosquitoes were identified morphologically in the field by use of a standardized key for the medically important anophelines of Southeast Asia (modified from IMPE [17]). Mosquitoes were individually stored in small tubes over silica gel for subsequent analyses.

### CSP-ELISA

All collected specimens were subjected to ELISA to detect the CSP of *P. falciparum *with monoclonal antibody Pf2A10-01, *P. vivax *210 with monoclonal antibody Pv-210-CDC and *P. vivax *247 with monoclonal antibody Pv-247-CDC in the head-thoracic portion of the mosquitoes [2-4]. All monoclonal antibodies were provided by CDC (Atlanta, USA). The ELISA was performed according to the protocol provided by MR4 [18]. In short, the capture monoclonal antibodies were bound to the plate, the well contents were aspirated and the remaining binding sites were blocked with blocking buffer (0.5% Casein technical, from bovine milk - Sigma, and 0.1N NaOH in PBS, pH 7.4). Mosquitoes to be tested were ground in blocking buffer containing IGEPAL CA-630, and an aliquot was tested. Positive controls (recombinant antigens provided by CDC, Atlanta, USA) and negative controls (laboratory reared female *Anopheles stephensi *mosquitoes prepared in the same way as the test samples) were tested for each plate. After an incubation of two hours at room temperature, the mosquito homogenate was aspirated and the wells were washed with PBS-Tween (0.05%). Peroxidase-linked monocolonal antibodies were then added to the wells. After 1 hour, the well contents were aspirated, washed again and the peroxidase substrate solution was added. After 30 minutes, the ELISA results (i.e. change in colour) were read visually and scored as 0 (no colour change, negative result), 1, 2, or 3, in which 1 was the lowest colour intensity and 3 was the highest colour intensity comparable with the positive control. Only 0 was considered as a negative result. It has to be noted that when using an ELISA-reader some specimens scored visually as 1 will fall below the cut-off value as specified by the MR4 protocol [18].

To check the heat stability of the antigen causing the false and true positivity, ten false positive specimens, thirteen true positive specimens, and six experimentally *P. falciparum *infected *Anopheles gambiae *s.s. mosquitoes (provided by the Malaria-Unit of UMC St Radboud, Nijmegen, the Netherlands) were re-analysed with the ELISA after heating the ELISA-homogenates in a heat block for 10 minutes at 100°C. The number of false positive specimens for which this could be done was limited because for most specimens not enough ELISA lysate was available after several rounds of retesting for confirmation of the ELISA results. The recombinant *P. falciparum *positive control protein used in the *P. falciparum *ELISA [19] was also checked for heat stability in different concentrations (2 pg/μl, 0.2 pg/μl and 0.02 pg/μl).

The abdomens of 19 *P. falciparum *true and 147 false positive specimens were subjected to *P. falciparum *ELISA as well.

### PCR detection of *Plasmodium *spp. or other parasites

#### DNA extraction

DNA extraction was performed on the ELISA-lysates by using the QIAcube (kit QIAamp DNA Micro Kit 56304, Qiagen) following the manufacturer's instructions. Briefly, 20 μl of the ELISA-lysate was added to 80 μl of ATL-buffer and extracted in the QIAcube machine, which eluted the DNA in 50 μl AE-buffer. One positive (i.e. a *P. falciparum *infected mosquito) and one negative control were included in every extraction run (10 test mosquitoes)

#### PCR

Before performing a *Plasmodium *specific PCR, every DNA extract was checked for the presence of mosquito DNA by the allele specific PCR for *Anopheles dirus *complex [20] or the ITS2 PCR for other mosquito species [21]. PCR specific for *Plasmodium *spp. was performed on all CSP-ELISA positive specimens and on all abdomens tested to confirm the presence of sporozoites by using primers PL1473F18 and PL1679R18 targeting the 18SrRNA [22]. For evaluation of the sensitivity of the CSP-ELISA technique, 299 CSP-ELISA negative *An. dirus *mosquitoes were tested by *Plasmodium *specific PCR.

The amplicons of PCR positive samples were cloned and sequenced for confirmation of the *Plasmodium *species (see below), or the samples were subjected to a more general Haemosporidia PCR [23] after which the amplicon could be sequenced directly. If the positive CSP-ELISA was not confirmed by *Plasmodium *specific PCR, this result was considered to be false positive. In this case, four PCR assays were used in order to detect related, possibly zoonotic, and vector-transmitted pathogens: Primers and PCR conditions of the PCR assays to detect parasites belonging to the Haemosporidia [23], to the Trypanosomatidae [24], to the Piroplasmida [25], and to the Haemogregarina [26] are given in Additional files 3 and 4 respectively.

#### Cloning

All amplicons were sequenced for final confirmation. Except for the Haemosporidia PCR assay, all amplicons were cloned before sequencing using the TOPO TA (Invitrogen K2000.01, Carlsbad, California) cloning kit following the manufacturer's instructions. The insert was amplified following the manufacturer's instruction and send to Genoscreen (Lille) for sequencing and sequence analysis. Sequence identification was performed through NCBI nucleotide-nucleotide BLAST searches [27].

#### PCR confirmation of previously analysed mosquitoes

CSP-ELISA positive mosquitoes previously reported [28] were also tested for PCR confirmation. Only specimens scoring 2 and 3 in the ELISA were considered as positive by Trung *et al *[28]. Until the PCR-analysis, these ELISA-homogenates have been stored at -80°C.

### Vector identification

The morphological species identification of the mosquitoes found positive for ELISA was confirmed by PCR using the PCR-RFLP for *Anopheles minimus *complex and *Anopheles pampanai *[29], and the allele specific PCR for *An. dirus *complex [20]. The identification of *Anopheles maculatus *was confirmed by sequencing (GenoScreen, Lille, France) the ITS2 rDNA region using primers ITS2A and ITS2B [21]. The sequences were blasted and compared with reference sequences [30]. No molecular identification was carried out for *Anopheles barbirostris, Anopheles jamesii *and *Anopheles splendidus*.

### Data analysis

For comparison of the false positivity between mosquitoes with a zoophilic trend and anthropophilic mosquitoes, a Chi square test was carried out using Epi Info 6. Based on the behavioural study of Trung *et al *[31] only *An. dirus *s.s. could be classified as anthropophilic, other vector species are regularly found on animals.

## Results

Specimens (N = 16,160) of seven anopheline species obtained by human landing collections were tested by CSP-ELISA for *P. falciparum *and *P. vivax*.

### CSP-ELISA on head-thorax portions and confirmation by *Plasmodium *spp. specific PCR

The results of the mosquito identification, CSP-ELISA and *Plasmodium *specific PCR are presented in Tables 2, 3 and 4. All specimens contained mosquito DNA as assessed by ITS-2 PCR or allele specific PCR for *An. dirus *complex. Only 21 out of 176 mosquitoes found positive with the *P. falciparum *CSP-ELISA could be confirmed by PCR (Table 2). The ELISA scores ranged between 1 and 3 for true positive and false positive specimens (Table 5). The Chi square test shows significantly more CSP-ELISA false positive mosquitoes in the anophelines that have a zoophilic trend as compared to the anthropophilic anopheline species, in Cambodia as well as in Vietnam (p < 0.001 for both tests). Sequence analysis of 20 out of 21 PCR positive specimens revealed the presence of *P. falciparum*; the remaining specimen (*An. barbirostris*) contained *Plasmodium malariae*.

**Table 2:** *P. falciparum *CSP-ELISA results and confirmation by *Plasmodium *spp. specific PCR

	Cambodia			Vietnam		
	
	N° Tested	N° ELISA +	N° PCR confirmed	N° Tested	N° ELISA +	N° PCR confirmed
**Anthropophilic**	**1144**	**12**	**11**	**864**	**12**	**8**

*An. dirus *s.l.	1144	12	11 ^1^	864	12	8 ^1^

**Zoophilic trend**	**8089**	**104**	0 (+1)*	**6063**	**48**	**1**

*An. barbirostris*	1628	33	0 (+ 1)*	na	na	na
*An. jamesii*	17	1	0	na	na	na
*An. maculatus *s.l.	3596	50	0	5081	42	0
*An. minimus *s.l.	2848	20	0	530	4	1 ^2^
*An. pampanai*	na	na	na	65	1	0
*An. splendidus*	na	na	na	387	1	0

The distinction into anthropophilic and zoophilic trend is made based on the results presented in Trung *et al*[31].

* The +1 mentioned between brackets means that the *Plasmodium *spp. specific PCR was positive, but cloning and subsequent sequencing of the PCR product revealed the presence of *P. malariae *instead of *P. falciparum*.

^1 ^All PCR confirmed specimens were molecularly identified as *An. dirus *s.s.

^2 ^The PCR confirmed specimen was molecularly identified as *An. minimus *s.s.

na = not applicable.

**Table 3:** *P.vivax *CSP-ELISA results and confirmation by *Plasmodium *spp. specific PCR

	Cambodia			Vietnam		
	
	N° Tested	N° ELISA +	N° PCR confirmed	N° Tested	N° ELISA +	N° PCR confirmed
**Anthropophilic**	**1144**	**5**	**4**	**864**	**4**	**4**

*An. dirus *s.l.	1144	5	4 ^1^	864	4	4 ^1^

**Zoophilic**	**8089**	**2**	**1**	**6063**	**6**	**3**

*An. barbirostris*	1628	1	0	na	na	na
*An. jamesii*	17	0	0	na	na	na
*An. maculatus *s.l.	3596	0	0	5081	5	2 ^2^
*An. minimus *s.l.	2848	1	1 ^3^	530	0	0
*An. pampanai*	na	na	na	65	1	1 ^4^
*An. splendidus*	na	na	na	387	0	0

The distinction into anthropophilic and zoophilic trend is made based on the results presented in Trung *et al *[31].

^1 ^All PCR confirmed specimens were molecularly identified as *An. dirus *s.s.

^2 ^All PCR confirmed specimens were molecularly identified as *An. sawadwongporni*

^3 ^The PCR confirmed specimen was molecularly identified as *An. minimus *s.s.

^4 ^The PCR confirmed specimen was molecularly identified as *An. pampanai*

na = not applicable.

**Table 4:** CSP-ELISA positive mosquitoes as reported in Trung *et al *[28] confirmed by *Plasmodium *spp. PCR

Village	Species	Number of mosquitoes positive by *Plasmodium *specific PCR/number of CSP-ELISA positive mosquitoes tested by PCR	
		
		*P. falciparum*	*P. vivax*
Lang Nhot (Khanh Hoa)	*An. minimus *	3/3	1/1
	*An. dirus *	1/1	0
Village 3 (Binh Thuan)	*An. dirus *	2/3 *	0
Cha Ong Chan (Rattanakiry)	*An. minimus *	1/1	0
	*An. dirus *	1/1 ^a^	0/1 ^a^

* The PCR negative sample gave a very weak signal when tested by mosquito specific ITS2 PCR, meaning that the DNA was not optimal anymore. This sample cannot be considered as CSP-ELISA false positive.

^a ^*P. falciparum *and *P. vivax *were detected by ELISA in the same mosquito. Sequencing of the PCR product only revealed *P. falciparum*.

**Table 5:** Number of true and false positive specimens scored as colour intensity 1, 2 or 3 for *P.falciparum *ELISA

	ELISA colour intensity score					
	
	Cambodia			Vietnam		
	
	1	2	3	1	2	3
**True positives**	**1**	**3**	**7**	**1**	**2**	**6**

*An. dirus *s.l.	1	3	7	1	2	5
*An. minimus *s.l.	0	0	0	0	0	1

**False positives**	**64**	**20**	**20**	**28**	**18**	**5**

*An. dirus *s.l.	1	0	0	4	0	0
*An. barbirostris*	7	8	17	na	na	na
*An. jamesii*	0	0	1	na	na	na
*An. maculatus *s.l.	38	10	2	23	15	4
*An. minimus *s.l.	18	2	0	1	2	0
*An. pampanai*	na	na	na	0	1	0
*An. splendidus*	na	na	na	0	0	1

na = not applicable.

In 12 out of 17 mosquitoes positive for the *P. vivax *CSP-ELISA, the presence of *Plasmodium *could be confirmed by PCR (Table 3). The ELISA results (colour intensity) of all non-confirmed specimens were scored as 1, whereas the results of confirmed specimens ranged between 1 and 3. Sequence analysis of all specimens positive by the *Plasmodium *specific PCR test revealed the presence of *P. vivax*.

CSP-negative mosquitoes from Cambodia were also checked by PCR on 299 specimens with well-conserved DNA. All of them were negative by *Plasmodium *specific PCR. Nine out of 18 CSP-ELISA positive samples from a previous study in Vietnam and Cambodia [28] were tested by *Plasmodium *spp. specific PCR (Table 4). All CSP-ELISA positive samples could be confirmed by the *Plasmodium *PCR and all but one were confirmed by sequencing: one *An. dirus *was positive for *P. falciparum *and *P. vivax *by ELISA, but sequencing confirmed the presence of *P. falciparum *only.

### Heat stability of the antigen causing the false positivity for the *P. falciparum *ELISA

Heating the ELISA homogenates at 100°C for 10 minutes, extracts of false ELISA positive specimens (N = 10) switched to a negative ELISA result, while the reaction remained positive with true ELISA positive field specimens (N = 13), experimentally infected mosquitoes (N = 6), and the recombinant positive control protein in the different concentrations tested (N = 3).

### PCR targeting other vector-transmitted parasites

By using the different PCR assays followed by sequencing of amplicons, no Haemosporida, Piroplasmida, Haemogregarina or Trypanosomatidae could be detected in the 160 CSP-ELISA false positive mosquitoes.

### CSP-ELISA on abdomens of positive specimens for the *P. falciparum *ELISA

Twelve out of 19 abdomens tested of *P. falciparum *ELISA true positive specimens were also positive for heat-stable CSP by ELISA and contained *Plasmodium *DNA. Three abdomens only contained *Plasmodium *DNA without CSP being detected in the abdomen, and four abdomens were negative for PCR and ELISA. Out of 147 abdomens tested of *P. falciparum *ELISA false positive specimens, 143 were negative for ELISA, and four were positive for the heat-unstable cross-reacting antigen (Table 6). None of these 147 abdomens contained *Plasmodium *DNA.

**Table 6:** ELISA results for the abdomens of specimens with true and false positive *P.falciparum *ELISA results of the head-thoracic portions

	Abdomens of *P. falciparum *ELISA true positive mosquitoes ^1^			Abdomens of *P. falciparum *ELISA false positive mosquitoes ^2^		
	**PCR +**	**PCR -**	**Total**	**PCR +**	**PCR -**	**Total**

ELISA score 0	3	4	**7**	0	143	**143**
ELISA score 1	5	0	**5**	0	3	**3**
ELISA score 2	3	0	**3**	0	1	**1**
ELISA score 3	4	0	**4**	0	0	**0**

**Total**	**15**	**4**	**19**	**0**	**147**	**147**

^1 ^For all abdomens of true positive mosquitoes, the ELISA score remained the same after heating the specimen for 10 minutes at 100°C.

^2 ^For all abdomens of false positive mosquitoes, the ELISA score turned to zero after heating the specimen for 10 minutes at 100°C.

## Discussion

The sporozoite rate is nowadays mainly estimated by using CSP-ELISA. Although some field studies, e.g. in Brazil [32] and Colombia [33], show a good concordance between the results of the CSP-ELISA and of the *Plasmodium *specific PCR, the present study shows that high rates of false positives can occur with the CSP-ELISA method, especially when tested for *P. falciparum *on vectors having a zoophilic biting trend present in Cambodia and Vietnam.

Although in the present study the source of the cross-reacting antigen was not identified, some light has been shed on the characteristics of this antigen: 1) it is mainly present in mosquitoes with a zoophilic biting trend; 2) it is heat-unstable; 3) it is mainly present in the head-thoracic portion of the mosquitoes and not in the abdomen; 4) all specimens containing the cross-reacting antigen were PCR negative for different parasites, namely the Haemosporida, Trypanosomatidae, Piroplasmorida and Haemogregarines.

The ELISA-method and reagents can be excluded as source of the false positivity as for every plate, all negative control mosquitoes always tested negative. As mentioned above, previous studies have reported false positivity in CSP-ELISA assays (Table 1). In literature, different hypotheses have been postulated about its source. A first hypothesis is a cross-reaction with a protein within the mosquito itself [12]. It has indeed been shown that the sera of rabbits immunized with the SPf66 malaria vaccine (containing the NANP-repeat of the CSP protein of *P. falciparum*) recognized several unidentified proteins of *Anopheles albimanus *[34]. However, if a constitutively expressed mosquito protein would be responsible for the reported false positivity, it would be observed systematically in a certain species, a higher number of false positives would be expected, and it would not explain the seasonal variation of the false positivity observed in South-Africa [12]. Therefore, if the cross-reacting antigen is a mosquito protein, it would be a protein that is expressed in reaction to a certain environmental or stress factor (e.g. immune proteins [35]), and only in the head-thoracic part of the mosquito.

A second hypothesis is a cross-reaction with an antigen in the blood of the host. Different sources of blood have been checked for their cross-reactivity in the CSP-ELISA: False positive results for *P. falciparum *and *P. vivax *have been found when testing the plasma fractions of pig and bovine blood in Thailand [15]. Also in Senegal, cows' blood reacted false positive in the CSP-ELISA used for detecting *P. malariae *and *Plasmodium ovale *sporozoites [13]. However, this false positivity was not systematically observed in all animals tested. Moreover, these factors are not stable in the blood circulation since some cows turned negative or weakly positive when examined 6 months later [13].

In most studies where false positive ELISA results were reported in mosquitoes [13,14,36,37] only head and thoraces were used for ELISA testing to exclude the contamination of oocyst-sporozoites present in the abdomen. False positives were associated with bovine and/or sheep blood meals when testing *An. gambiae *s.l for the presence of *P. malariae *and *P. ovale *in Senegal [13] and when testing *Anopheles parensis *for the presence of *P. falciparum *in South-Africa [14]. However, it has been assumed that the blood that remains in the pharynx of the mosquito is too few to result in a false positive reaction [15]. In the present study, mosquitoes were collected by human landing collection, thus the great majority being unfed (> 95%). Moreover, fed proteins can circulate in the hemocoel [38] and should, therefore, also be present in the abdomen of the mosquito, which was not the case for the cross-reacting antigen in the present study. This thus excludes (animal) host antigens from being the source of the cross reaction, unless for some unknown reason they would remain or be concentrated in the head-thoracic part of the mosquito.

Because of the mentioned association with bovine and swine blood [13-15], and because of the presence of the false positivity in mainly zoophilic anophelines, the present study explored the possibility of other parasites, possibly transmitted by vectors and/or originating from animals, cross-reacting in the CSP-ELISA. This study has confirmed that the false positive reaction in the *P. falciparum *and *P. vivax *CSP-ELISAs is certainly not caused by another *Plasmodium *sp. Only one ELISA positive sample contained a different *Plasmodium *species, namely *P. malariae*. The 160 false positive specimens did not contain *Plasmodium *species. Two different PCR targets were used to detect *Plasmodium *species in the mosquitoes, which excludes the possibility of primer binding site mutation that would result in a false negative PCR. Indeed primer binding site mutation in two targets would be very unlikely, especially in the well conserved mitochondrial genome of *Plasmodium *spp. [23]. It has been previously stated that false positivity is caused by the CSP antigen of sporozoites before their invasion of the salivary glands or after an abortion of the infection [8,9]. However, in the false positive specimens reported in this study oocyst sporozoites were absent, as the abdomens were negative for heat-stable CSP in ELISA and for *Plasmodium *DNA. Moreover this would not explain the fact that mostly zoophilic mosquitoes react false positive. As the recombinant control protein contains only the heat-stable repeat region, a digested CSP is also not a likely cause of the false positivity.

This study also excludes parasites belonging to the Haemogregarina, Piroplasmida or Trypanosomatidae as causes of the false positivity, within the detection limits of the PCR assays used. These parasites were chosen as targets because of their taxonomic proximity to *Plasmodium *or the fact that they can be transmitted by vectors. Additionally, there is a number of parasites that are too big to be taken up by mosquitoes (e.g. *Schistosoma *spp. [39] or *Angiostrongylus vasorum *[40]) or that do not reside in the blood of their host (eg *Toxoplasma *sp. [41]). Blood circulating antigens of these parasites taken up by the mosquito during a blood meal, and not detectable by PCR, could induce a false positive reaction in the CSP-ELISA. However, there is no explanation for why this kind of antigens would remain or be concentrated in the head-thoracic part of the mosquito.

In India [42], Papua New Guinea [43], Bangladesh [44], and Vietnam [45], recent studies suggest the involvement of several vector species in the transmission of malaria based on CSP-ELISA results only. In these publications, most of the authors discuss the possibility of false positivity in their results [42-44], but no attempts have been made for confirming the results, or the possibility has been discarded because of the absence of cattle in the study area [43]. The present study shows that false positivity can be considerably high in some of these species (e.g. *An. barbirostris, An. maculatus *and *An. minimus *s.l.). However, transmission data of a previous study in Cambodia and Vietnam by Trung *et al *[28] were confirmed.

Present study clearly confirmed *An. dirus *s.s. as the main vector of malaria in Vietnam and Cambodia. The role of *An. minimus *s.l., *An. maculatus *s.l. and *An. barbirostris *in *P. falciparum *malaria transmission is clearly overestimated when considering only non-confirmed CSP-ELISA results. *Anopheles pampanai *was for the first time confirmed as vector of *P. vivax *in Vietnam [16], and *An. barbirostris *as vector of *P. malariae *in Cambodia. False positives were mainly observed with *P. falciparum *CSP-ELISA, while this was less the case with the *P. vivax *assay. With the *P. falciparum *ELISA assay, one positive specimen showed the presence of *P. malariae *instead of *P. falciparum*. Alignment of the amino-acids of their respective repeat regions (given in Additional file 5) shows that it is probably not due to a cross-reaction between the *P. malariae *repeat (NAAG/NDAG [46]) and the monoclonal antibody against the *P. falciparum *repeat (NANP/NVDP [46]). However, it is possible that the Haemosporida PCR favours the amplification of one *Plasmodium *species when more than one species is present in the mosquito. This could also be the case for the *An. dirus *specimen positive in ELISA for both *P. falciparum *and *P. vivax *in which sequencing confirmed the presence of *P. falciparum *only (Table 4).

## Conclusion

The CSP-ELISA can considerably overestimate the EIR, particularly for *P. falciparum *transmission and when dealing with zoophilic species. Although the cross-reacting antigen remains unknown, this study has shown that it is heat-unstable and can be removed by heating the ELISA lysate (100°C, 10 min). It is, therefore, highly recommended to confirm all positive CSP-ELISA results by a second CSP-ELISA test on the heated ELISA lysate, especially in anophelines with a known zoophilic trend. Confirmation by the *Plasmodium *specific PCR followed if possible by sequencing of the amplicons for *Plasmodium *species determination is also recommended, although PCR is not stage specific for sporozoites.

## Competing interests

The authors declare that they have no competing interests.

## Authors' contributions

LD, MC en WVB designed the study. WVB and LD supervised the work critically, LD carried out the data analysis and drafted the manuscript. HDT and TS facilitated and supervised the field work, and critically reviewed the manuscript. LED and PR carried out the ELISA assays and molecular identification of the collected mosquitoes and critically reviewed the manuscript. AV carried out the PCR assays and critically reviewed the manuscript. MC critically reviewed the manuscript. All authors read and approved the final manuscript.

## Supplementary Material

Additional file 1 **Study villages in Cambodia**. The data provide a summarized description of the twelve forested study villages in Cambodia. Click here for file

Additional file 2 **Study villages in Vietnam**. The data provide a summarized description of the eight study villages in Vietnam. Click here for file

Additional file 3 **Primers for parasite detection**. The data provided represent the primers used in the different PCR assays for parasite detection. Click here for file

Additional file 4 **PCR conditions for parasite detection**. Details are provided for the PCR reaction mix and PCR cycling conditions for the different PCR assays for parasite detection. Click here for file

Additional file 5 **CSP repeat region alignment of *P. malariae *and *P. falciparum***. Alignment of the amino-acids sequences of the repeat regions of *P. malariae*, and *P. falciparum *with respective Pubmed Accession Numbers AAA29557 and AAA29554. Click here for file
