# Supplementary material for: False positive circumsporozoite protein ELISA: a challenge for the estimation of the entomological inoculation rate of malaria and for vector incrimination
Source: Malar J. 2011 Jul 18;10:195. doi: 10.1186/1475-2875-10-195 (PMC3160429; doi:10.1186/1475-2875-10-195)
Supplement: Additional file 1 — Study villages in Cambodia. The data provide a summarized description of the twelve forested study villages in Cambodia. [file 1475-2875-10-195-S1.DOC]

| **Province** | **Commune** | **Village** | **Elevation**  **(m)** | N° DD MM SS | E° DD MM SS | Environment |
| --- | --- | --- | --- | --- | --- | --- |
| Rattanakiri | Samaki | Bornhuk2 | 303 | 13°48’12” | 107°06’03” | Rubber plantation |
| Rattanakiri | Samaki | Ping | 259 | 13°49’47” | 107°05’48” | Scattered |
| Rattanakiri | Samaki | Prac | 263 | 13°49’35” | 107°05’45” | Ever green forest |
| Rattanakiri | Kok | Sala | 247 | 13°42’50” | 107°14’47” | Deforested environment |
| Rattanakiri | Kok | Leutouch | 218 | 13°43’53” | 107°15’14” | Village with forest plots: 70% forest |
| Rattanakiri | Kechong | Saleo | 238 | 13°44’07” | 107°14’08” | Forest kamka |
| Pursat | Promoy | Tang Yo |  | 12°23’06” | 103°16’38” | Deforested Ever green/ deciduous. |
| Pursat | Promoy | Don Neak | 259 | 12°22’00” | 103°13’27” | Deciduous/deforested |
| Pursat | Anlong Reap | Dey Krahorm Leu | 337 | 12°16’26” | 102°57’08” | Evergreen/deforested |
| Pailin | Otavao | O-Kting | 187 | 12°46’40” | 102°42’05” | Evergreen/forest (not visited) |
| Pailin | Otavao | Pang Rolim | 200 | 12°47’17” | 102°41’28” | Deciduous/deforested |
| Pailin | Steung kach | Tick Cheng | 147 | 12°55’20” | 102°40’43” | Evergreen/deforested |

Additional file 1: Summarized description of the twelve forested study villages in Cambodia
