# Supplementary material for: False positive circumsporozoite protein ELISA: a challenge for the estimation of the entomological inoculation rate of malaria and for vector incrimination
Source: Malar J. 2011 Jul 18;10:195. doi: 10.1186/1475-2875-10-195 (PMC3160429; doi:10.1186/1475-2875-10-195)
Supplement: Additional file 2 — Study villages in Vietnam. The data provide a summarized description of the eight study villages in Vietnam. [file 1475-2875-10-195-S2.DOC]

| **Province** | **Commune** | **Village** | **N° DD MM SS** | **E° DD MM SS** |
| --- | --- | --- | --- | --- |
| Ninh Thuan | MA NOI | HA ZAI | 11° 39' 07" | 108° 41' 52 " |
| Ninh Thuan | MA NOI | TA NOI | 11° 37' 45" | 108° 38' 47" |
| Ninh Thuan | MA NOI | THON DO | 11° 39' 35 | 108° 42' 31" |
| Ninh Thuan | PHUOC BINH | BAC RAY 1 | 12° 00' 81" | 108° 48' 93" |
| Ninh Thuan | PHUOC BINH | BAC RAY 2 | 12° 00' 08" | 108° 48' 75" |
| Ninh Thuan | PHUOC BINH | BO LANG | 12° 00' 09" | 108° 47' 35" |
| Ninh Thuan | PHUOC BINH | GIA E | 11° 59' 56" | 108° 46' 59" |
| Ninh Thuan | PHUOC BINH | HAN RAC 1 | 11° 58' 54" | 108° 45' 19" |

Additional file 2: Summarized description of the eight study villages in Vietnam
