# Supplementary material for: False positive circumsporozoite protein ELISA: a challenge for the estimation of the entomological inoculation rate of malaria and for vector incrimination
Source: Malar J. 2011 Jul 18;10:195. doi: 10.1186/1475-2875-10-195 (PMC3160429; doi:10.1186/1475-2875-10-195)
Supplement: Additional file 3 — Primers for parasite detection. The data provided represent the primers used in the different PCR assays for parasite detection. [file 1475-2875-10-195-S3.DOC]

**Additional file 3**: Primers for parasite detection

| Target group | Primers | Primer sequences | Reference |
| --- | --- | --- | --- |
| *Plasmodium* spp | PL1473F18 | 5’ TAACGAACGAGATCTTAA 3’ | [22] |
|  | PL1679R18 | 5’ GTTCCTCTAAGAAGC TTT 3’ | [22] |
| Haemosporida | PLAS-1 | 5’ GAGAATTATGGAGTGGATGGTG  3’ | [23] |
|  | PLAS-2 | 5’ GTGGTAATTGACATCCWATCC 3’ | [23] |
|  | PLAS-3 | 5’ GGTGTTTYAGATAYATGCAYGC 3’ | [23] |
|  | PLAS-4 | 5’ CATCCWATCCATARTAWAGCATAG 3’ | [23] |
| Trypanosomatidae | 18S-F | 5’ CGCCAAGCTAATACATGAACCAA 3’ | [24] |
|  | 18S-R | 5’ TAATTTCATTCATTCGCTGGACG 3’ | [24] |
| Piroplasmorida | PYRO-F1 | 5’ GCATTTAGCGATGGACCATTCAAG 3’ | [25] |
|  | PYRO-F2 | 5’ GCCGGCGATGTATCATTCAAG 3’ | [25] |
|  | PYRO-R | 5’ CCTGTATTGTTATTTCTTGTCACTACCTC 3’ | [25] |
| Haemogregarines | HEMO-1 | 5’ TATTGGTTTTAAGAACTAATTTTATGATTG 3’ | [26] |
|  | HEMO-2 | 5’ CTTCTCCTTCCTTTAAGTGATAAGGTTCAC 3’ | [26] |
