# Supplementary material for: False positive circumsporozoite protein ELISA: a challenge for the estimation of the entomological inoculation rate of malaria and for vector incrimination
Source: Malar J. 2011 Jul 18;10:195. doi: 10.1186/1475-2875-10-195 (PMC3160429; doi:10.1186/1475-2875-10-195)
Supplement: Additional file 4 — PCR conditions for parasite detection. Details are provided for the PCR reaction mix and PCR cycling conditions for the different PCR assays for parasite detection. [file 1475-2875-10-195-S4.DOC]

**Additional file 4**: PCR conditions

| PCR | Cycling Program | PCR reaction mix | | | | | | |
| --- | --- | --- | --- | --- | --- | --- | --- | --- |
|  |  | Total reaction volume (µl) | Taq (U) | dNTP (pmol/µl) | Primers (pmol/µl) | MgCl_2_ (mmol/l) | BSA (mg/ml) | DNA extract (µl) |
| *Plasmodium* spp | 94°C, 15 min  35 x (94°C, 60s; 50°C, 60s; 72°C, 120s)  72°C, 5 min | 50 | 1 | 200 | 25 | 5 |  | 5 |
| Haemosporida | 94°C, 5 min  40 x (94°C, 30s; 55°C, 30s; 72°C, 90s)  72°C, 10 min | 25 | 2.5 | 200 | 25 | 2 |  | 2.5 |
| Trypanosomatidae | 94°C, 15 min  30 x (94°C, 30s; 60°C, 30s; 72°C, 30s)  72°C, 5 min | 25 | 0.5 | 200 | 0.8 | 2.5 | 0.1 | 2.5 |
| Piroplasmorida | 94°C, 5 min  35 x (94°C, 60s; 63°C, 60s; 72°C, 60s)  72°C, 5 min | 25 | 0.625 | 200 | 12.5 |  |  | 2.5 |
| Haemogregarines | 94°C, 15 min  40 x (94°C, 60s; 54°C, 60s; 72°C, 60s)  72°C, 5 min | 25 | 0.625 | 200 | 0.8 | 5 | 0.1 | 2.5 |
