# Supplementary figures and images for: False positive circumsporozoite protein ELISA: a challenge for the estimation of the entomological inoculation rate of malaria and for vector incrimination
Source: Malar J. 2011 Jul 18;10:195. doi: 10.1186/1475-2875-10-195 (PMC3160429; doi:10.1186/1475-2875-10-195)

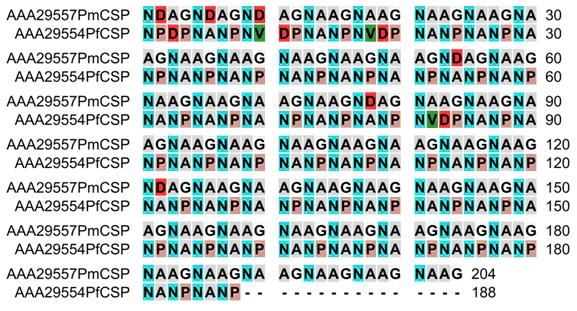

Supplement: Additional file 5 — CSP repeat region alignment of P. malariae and P. falciparum. Alignment of the amino-acids sequences of the repeat regions of P. malariae, and P. falciparum with respective Pubmed Accession Numbers AAA29557 and AAA29554. [file 1475-2875-10-195-S5.JPEG]
